# Attitudes in China about Crops and Foods Developed by Biotechnology

**DOI:** 10.1371/journal.pone.0139114

**Published:** 2015-09-29

**Authors:** Fei Han, Dingyang Zhou, Xiaoxia Liu, Jie Cheng, Qingwen Zhang, Anthony M. Shelton

**Affiliations:** 1 Department of Entomology, China Agricultural University, Beijing, P. R. China; 2 College of Resources Science and Technology, Beijing Normal University, Beijing, P. R. China; 3 Department of Entomology, Cornell University/New York State Agricultural Experiment Station (NYSAES), Geneva, New York, United States of America; 4 Institute of Population and Labor Economics, Chinese Academy of Social Sciences, Beijing, P. R. China; University of Vermont, UNITED STATES

## Abstract

Transgenic Bt cotton has been planted in China since 1997 and, in 2009, biosafety certificates for the commercial production of Bt rice and phytase corn were issued by the Chinese government. The public attitude in China toward agricultural biotechnology and genetically modified (GM) crops and foods has received considerable attention worldwide. We investigated the attitudes of consumers, Bt cotton farmers and scientists in China regarding GM crops and foods and the factors influencing their attitudes. Data were collected using interview surveys of consumer households, farmer households and scientists. A discrete choice approach was used to elicit the purchase intentions of the respondents. Two separate probit models were developed to examine the effect of various factors on the choices of the respondents. Bt cotton farmers had a very positive attitude because Bt cotton provided them with significant economic benefits. Chinese consumers from developed regions had a higher acceptance and willingness to pay for GM foods than consumers in other regions. The positive attitude toward GM foods by the scientific community will help to promote biotechnology in China in the future. Our survey emphasized that educational efforts made by government officials, the media and scientists can facilitate the acceptance of GM technology in China. Further educational efforts will be critical for influencing consumer attitudes and decisions of government agencies in the future. More effective educational efforts by government agencies and public media concerning the scientific facts and safety of GM foods would enhance the acceptance of GM crops in China.

## Introduction

Genetic modification (GM) of organisms can provide novel and beneficial traits in plants, animals and microorganisms [1]. The use of genetic engineering (GE) has been especially rapid in agriculture [2–5]. Transgenic crops have been developed for resistance to insects, diseases, environmental stresses and provided improved quality and yield and better weed management [6–9].

Cultivation of genetically modified (GM) crops has also increased income to farmers [10, 11], especially in resource-poor regions [12]. GM crops can help solve the increasingly serious conflict between population growth and environmental resources [10, 11, 13, 14]. From 1996 to 2012, data indicate the cumulative gain of global GM crops was $ 133 billion, while the total savings of pesticides was 497 million kg [14]. The global area of GM crops was 181.5 million hectares in 2014, which has increased more than 100-fold compared with 1.7 million in 1996. In 2014, GM crops were grown in 28 countries with the six largest plantings in the US, Brazil, Argentina, India, Canada, and China [14].

The most successful commercialization of a GM crop in China is cotton that produces insecticidal crystal (Cry) proteins from the bacterium, *Bacillus thuringiensis* (Bt), which has significant resistance to lepidopteran pests [15]. First planted in China in 1997, the planting area of Bt cotton was 3.9 million hectares in 2014, which was 93% of the total cotton production in China, and planted by 7.1 million small-scale farmers [14]. Food security has become an important political issue in China because the declining increases in crop yields will not meet the needs of a growing population. Use of genetic engineering has been recognized as a way of promoting the sustainable development of Chinese agriculture. Therefore, in 2008 the Chinese State Council launched a major project with $US 3.5 billion to support crop improvement using transgenic crops [16].

In 2009, the Chinese Ministry of Agriculture approved the bio-security certificates of Bt rice and phytase maize [2, 17]. However, these two GM crops have not been promoted for commercial production. Public attitudes on GM crops and foods will ultimately determine whether GM crops and foods are grown and whether they will be accepted in the marketplaces in China. Studies have investigated the public attitude towards GM foods and crops in the European Union and US [18–22], while there have been limited studies on the Asian attitude towards GM foods [23], with only a few reports published on the attitude of consumers toward GM foods in China [24–27].

Although Chinese farmers have been growing a GM crop (cotton) since 1997, there have been few studies assessing the attitude of Chinese farmers on the long-term planting of transgenic crops, and no studies have reported the attitude of Chinese scientists toward GM crops. The aim of this research was to fill this gap by examining the views of the important stakeholder groups regarding GM crops and foods, including consumers, producers (farmers), and scientists (academia). In this study we also discuss the impacts of the stakeholders’ decisions on the future of transgenic crops and foods and policy-making in China.

## Results

### The majority of Chinese consumers would accept GM foods

**Fig 1:**
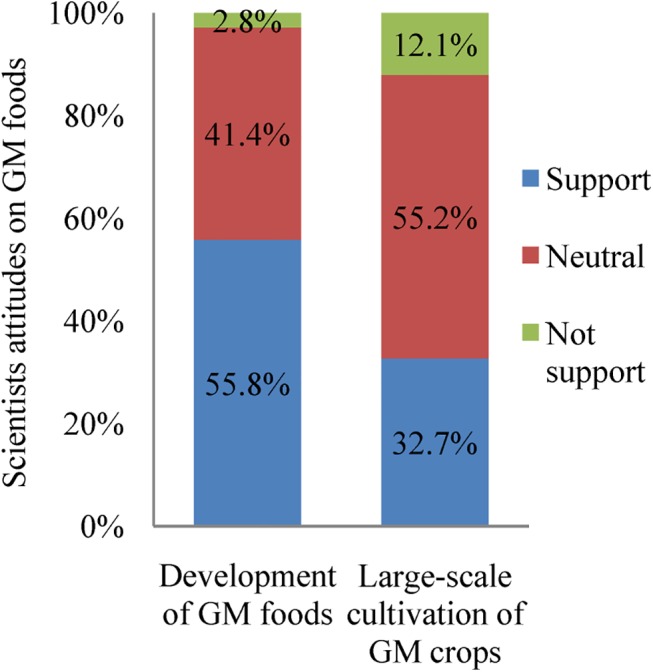
The attitudes of Chinese consumers about GM foods (2007–2008, 2010). There were 1,416 and 1,759 respondents in 2007–2008 and 2010, respectively. (A) Attitude of consumers whether GM foods should be sold, (B) Percentage of consumers who believed they had purchased GM foods.

The first survey was conducted from 2007 to 2008, and the second survey was conducted in 2010. The options for the choices of consumers for GM foods were: support, follow the recommendations of the government, undecided, or do not support. The results were 21.9%, 28.2%, 23.7%, and 26.2% of the consumers, respectively, in the first survey and 18.1%, 15.2%, 34.6%, and 32.1% of the consumers, respectively, in the second survey (Fig 1A). Removing the consumers who do not support the sales of GM foods, suggests that 73.8% and 67.5%, respectively, of the consumers in each surveys may be accepting of the sale of GM foods. These figures represent a large potential consumer group. The percentage of consumers with no knowledge of GM foods was 19.8% in the first survey and 6.0% in the second survey (Table 1), indicating an increase in Chinese consumers’ awareness of GM foods. Consumers who believed they had purchased or never purchased GM food was 33.0% and 30.9%, respectively, from 2007 to 2008, whereas these figures were 43.3% and 19.2%, respectively, in 2010 (Fig 1B). These results revealed that approximately 10% more people believed they had purchased GM foods in the later survey.

**Table 1:** Attitudes, knowledge and sources of information for Chinese consumers on GM foods (2007–2008, 2010).

Interviewees' response	Year (total number of interviewees)	Percentage responding to each category						
1. Awareness of GM foods								
		Very familiar	Understand a little	Know the basics	No knowledge			
	2007–2008, (1,416)	5.30%	46.60%	28.30%	19.80%			
	2010, (1,759)	6.30%	35.50%	52.20%	6.00%			
2. Main ways of learning about GM foods ^1^								
		TV/radio	Print media	Internet	Introduction by family or friends	Street or store promotion	Work or study	Other
	2007–2008, (1,416)	50.00%	26.30%	15.80%	15.60%	6.00%	15.20%	7.70%
	2010, (1,759)	51.10%	27.70%	22.10%	17.60%	6.50%	28.50%	1.90%
3. Considered to provide credible and fair information about GM foods ^1^								
		TV/radio	Print media	Internet	Introduction by family or friends	Street or store promotion	Work or study	Other
	2010, (1,759)	52.00%	24.50%	8.00%	7.10%	2.20%	29.20%	2.70%
4. Weight on environmental or economic factor ^2^								
		1	2	3	4	5		
	2010, (1,759)	56.30%	17.00%	21.90%	2.30%	2.50%		

^1^ This variable was a multiple-choice test question.

^2^ When consumers were asked about the importance of different factors in agriculture, a value of 1 indicated that protection of the environment was important whereas a value of 5 indicated that economic development was more important.

Chinese consumers gained knowledge of GM foods from diverse media, including TV, radio, print media, internet, and others (Table 1). TV together with radio was the main method (50.0 and 51.1% in the two surveys) by which Chinese consumers gained knowledge of GM foods, followed by print media (26.3 and 27.7% in the two surveys). TV together with radio was considered to be the most credible and fair sources of information (Table 1). These results revealed that the media can play a significant role in advancing or discouraging consumer acceptance of GM crops and foods.

**Fig 2:**
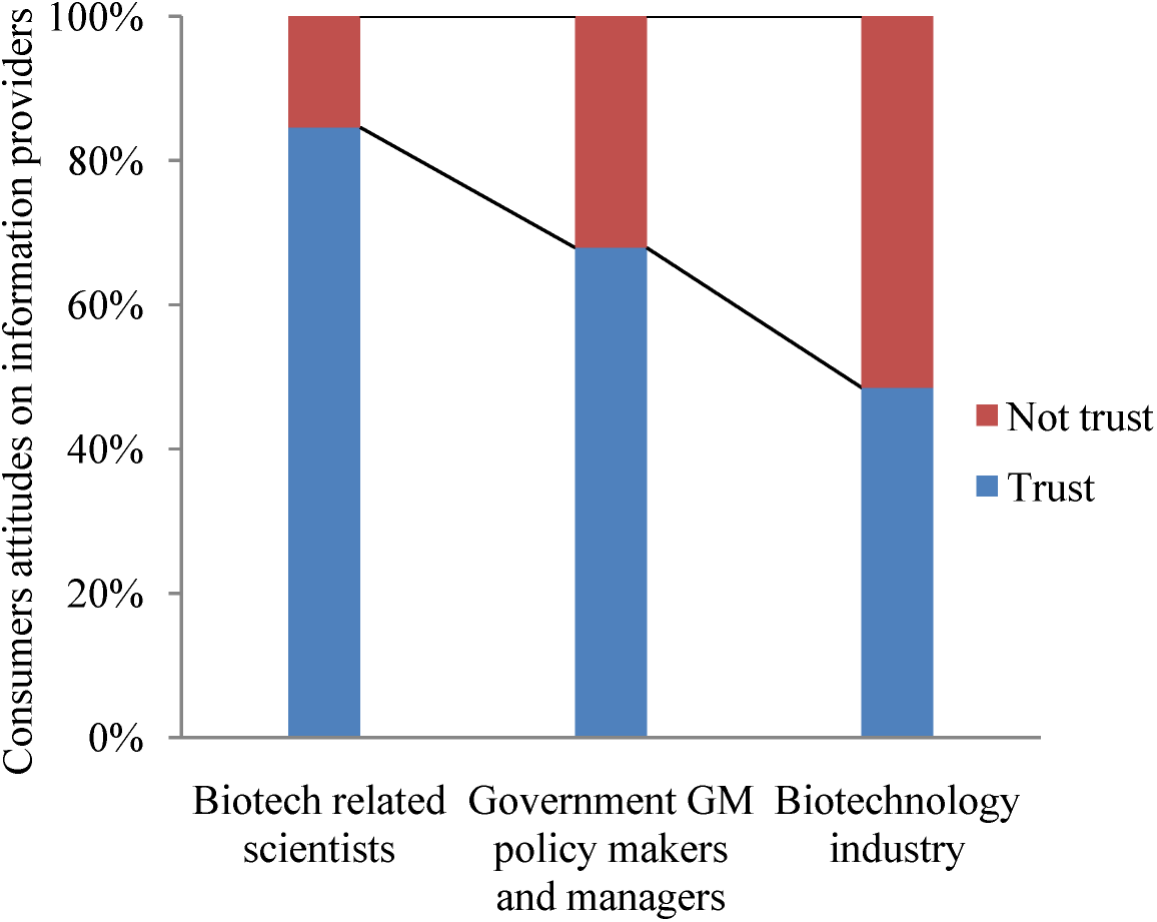
The attitudes of Chinese consumers about providers of information about GM crops and food (2010). There were 1,759 respondents.

The attitudes of consumers on different types of GM products were also analyzed in 2010. Opposition (including strong opposition) to non-edible products of GM crops, edible products of GM crops, transgenic animal products, transgenic fish, and GM (Bt) rice was 5.2%, 22.2%, 38.6%, 39.7%, and 18.3% of consumers, respectively (Table 2). These results indicated that a majority of Chinese consumers would not be opposed to different types of GM products. For those who would accept GM products, the highest acceptance rates were for non-edible products of GM crops (73.9%) followed by GM (Bt) rice (50.6%). Only 28.6% of Chinese respondents said they would not purchase insect-resistant GM (Bt) rice (Table 2), suggesting this important food product could have high acceptance in the Chinese market. Our survey also indicated that Chinese consumers trusted scientists (84.6%) and the government policy makers and managers (67.9%) on matters concerning biotechnology, but had less trust in the biotechnology industry (48.5%; Fig 2). On matters involving GM, survey results indicated that Chinese consumers considered protection of the environment to be more important than economic development (Table 1).

**Table 2:** Acceptance of Chinese consumers for different GM foods (2010).

Interviewees' response	Percentage responding to each category					
	Fully accepted	Accepted	Neutral	Opposed	Strongly opposed	Do not know
Non-edible products of GM crops	32.3%	41.6%	19.2%	3.9%	1.3%	1.7%
Edible products of GM crops	8.5%	34.7%	32.9%	17.1%	5.1%	1.7%
Transgenic animal products	4.4%	18.8%	34.8%	28.4%	10.2%	3.4%
Transgenic fish	4.7%	17.7%	33.9%	27.9%	11.8%	4.0%
GM-Bt rice	12.4%	38.2%	28.4%	14.4%	3.9%	2.7%
	Fully Willing	Willing	Neutral	Unwilling	Strongly unwilling	Do not know
Willingness to pay for GM-Bt rice	8.1%	33.9%	29.4%	22.7%	5.9%	0

### Chinese farmers and scientists have a positive attitude toward GM foods

**Fig 3:**
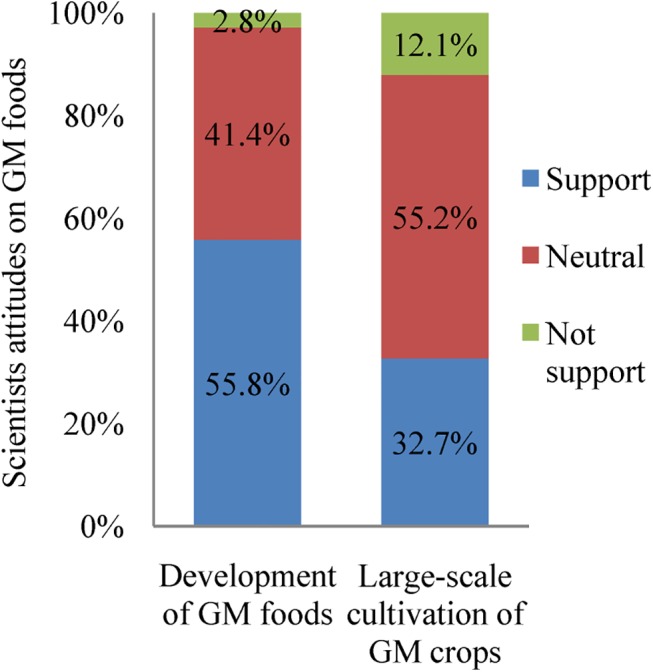
The attitudes of Chinese scientists about GM foods and large-scale production of GM crops (2007–2008). There were 254 respondents.

The attitudes of Chinese farmers and scientists toward GM foods were surveyed in our first study (2007–2008). The results showed that 83.8% of farmers planting Bt cotton had a long-term willingness to plant Bt cotton and 87.3% supported the production of GM plants in the future (Table 3). These results suggest that most Chinese farmers have a positive attitude toward the future cultivation of GM crops. Furthermore, education regarding Bt plants by local agricultural extension workers (56.9%) and neighbors (26.1%) were the main methods for conveying information regarding Bt cotton technology to farmers (Table 3). Only 2.8% and 12.1% of the academicians, the members of China’s highest scientific bodies, did not support the development of GM food and the large-scale cultivation of GM crops, respectively (Fig 3). The majority (55.8%) support development of GM foods. A large proportion (41.4 and 55.2%) were neutral on both questions.

**Table 3:** Attitudes of Chinese farmers about GM foods (2007–2008).

Interviewee response	Percentage responding to each category			
1. Willingness to plant Bt cotton				
	Long-term cultivation	Short-term cultivation	Continue not to grow	Undecided
	83.80%	13.00%	0.80%	2.40%
2. Attitude about distributing GM plants in China in the future				
	Support	Support if helpful to make money	Do not support	Undecided
	60.70%	26.60%	1.20%	11.50%
3. Main method of learning about Bt cotton technology				
	Neighbors	TV	Print media	Local agricultural extension workers
	26.10%	12.70%	4.30%	56.90%

### Multiple factors influence the choice of Chinese consumers

The model estimate for consumers’ willingness to buy GM foods identifies the relationships among the respondents' perceptions concerning GM foods and their socioeconomic and value attributes (Table 4). According to our first survey, carried out from 2007 to 2008, knowledge of GM foods played an important role in influencing consumers’ purchasing behavior of GM foods, as indicated by the coefficients of “*Awareness*” and “*Attitudes*” being highly significant. The data showed that Chinese consumers in developed regions (the Eastern, Central and Northeast regions) had a higher acceptance and willingness to purchase GM foods than Chinese consumers in less-developed areas (the Western region). The significantly positive coefficient on the “*Income*” variable illustrated that consumers' willingness to purchase GM foods was stronger when the respondents had a higher income level (Table 4). These are the first data on this topic reported from China. “*Awareness*” and “*Attitudes*” of GM foods in this study were significant variables, indicating that Chinese consumers who have a higher level of awareness of GM foods are more likely to buy them. The majority of polled Chinese consumers have some basic knowledge of GM foods, but the cognitive level is not high.

**Table 4:** Analysis of factors influencing consumers’ willingness to purchase GM foods from 2007 to 2008 and 2010. Two binary probit models were used to estimate the model coefficients. Source: Authors’ survey.

Probit model in 2007 to 2008				Probit model in 2010			
Independent variables	Mean	Coefficient	z-Statistic	Independent variables	Mean	Coefficient	z-Statistic
Awareness	2.81±0.81	0.4071***	8.5054	Awareness	2.42±0.70	0.3953***	8.4790
Attitudes	2.58±1.12	0.3067***	4.0857	Attitudes	0.33±0.47	0.2181***	3.3051
Ln (Income)	0.83±0.56	0.2874***	3.6393	Ln(Income)	8.46±0.65	0.0897*	1.7887
Gender	0.51±0.5	-0.1467*	-1.9334	Gender	0.51±0.50	-0.1377**	-2.2080
Age	34.66±12.87	-0.0012	-0.3718	Age	33.42±13.21	0.0045*	1.8936
Eastern Region	0.59±0.49	0.3784***	3.7773	Eastern Region	0.30±0.46	0.2286***	2.7569
Central Region	0.14±0.35	0.3102**	2.3233	Central Region	0.27±0.44	0.2823***	3.2747
Northeast	0.15±0.36	0.6164***	3.4249	Northeast	0.16±0.37	0.6319***	6.5096
Intercept		-1.8639***	-9.8330	Intercept		-2.2946***	-5.1945
Log likelihood = —771.5402 McFadden R-squared = 0.0807				Log likelihood = -1128.741 McFadden R-squared = 0.0584			

Values are the means ± SD. The symbols *, ** and *** denote significance at *P* = 10%, 5% and 1% level, respectively.

The model estimate of the second survey in 2010 confirmed the previous results (Table 4). Consumer acceptance and awareness of GM foods led to decisive actions to purchase them. The purchasing behaviors of consumers were significantly different in different regions of China, especially consumers living in the western regions. “*Gender*” and “*Age*” were significant variables (Table 4), indicating the purchase intention of older individuals and females was stronger.

### Economic benefits strongly influence the choice of Chinese farmers

“*Family-income change*” was a highly significant factor in the estimate of farmers' willingness to plant Bt cotton (Table 5). Additionally, number of years farmers had planted and the area on which they planted Bt cotton were significant factors in farmers’ decisions for long-term cultivation of Bt cotton. A similar positive relationship was observed in the proportion of agricultural income to total household income. Farmers were more concerned with the long-term benefits of increasing production and their income by planting Bt cotton than reducing pesticide use (no statistical significance for this coefficient; Table 5). Based on our model estimation, the economic benefits of GM crops strongly influenced the behavior of Chinese farmers. Farmers from the Yellow River Basin and Yangtze River Basin were more supportive of the long-term planting of Bt cotton than those in Xinjiang. The adoption of Bt cotton in Xinjiang is still very limited because of its later introduction and fewer available Bt cotton varieties compared to other regions. Our data suggest that farmers’ experience with planting Bt cotton was an important factor that influenced their farming practices, although this factor is often associated with long-term economic interests.

**Table 5:** Analysis of factors influencing farmers’ long-term willingness to plant Bt cotton from 2007 to 2008. A binary probit model was used to estimate the model coefficients. Source: Authors’ survey.

Independent variables	Mean	Coefficient	z-Statistic
Cultivating area (ha)	2.05±2.82	0.5330***	4.5935
Age	42.86±8.89	0.0147*	1.8336
Fields proportion (%)	67.98±31.62	0.0016	0.4897
Agri-income proportion (%)	2.25±0.78	-0.2720***	-2.8444
Years of cultivation	2.78±0.70	-0.5131***	-4.6237
Gender	0.86±0.35	-0.7007 ***	-3.1013
Education level	3.12±0.71	-0.0058	-0.0555
Family-income change (RMB)	4623.83±5289.72	0.0001 ***	3.4514
Pesticide change (kg/ha)	-21.58±31.56	-0.0028	-0.9397
Production change (kg/ha)	804.84±686.85	0.0004**	2.9993
Artificial-input change	-140.66±140.05	0.0003	0.4617
Yellow-River Basin	0.50±0.50	0.9676 ***	3.0508
Yangtze-River Basin	0.11±0.31	1.0735**	2.3175
Intercept		1.1939	1.4084
Log likelihood = -203.2356 McFadden R-squared = 0.3808			

Values are the means ± SD. The symbols *, ** and *** denote significance at *P* = 10%, 5% and 1% level, respectively.

## Conclusions and Policy Implications

Several important GM crops and foods have been developed for the Chinese market [2, 14, 16], but their success depends on the attitudes of several stakeholder groups. Previous surveys focused on the attitudes of Chinese consumers [24–27]. The present study documented the views of three key stakeholder groups in China, including consumers, farmers, and scientists.

In the process of developing a safer and more abundant food supply through biotechnology, the Chinese government has been focusing on educational issues for farmers and consumers. This focus seems reasonable if China is to meet the food requirements of its growing population. Chinese policymakers generally make their decisions regarding agricultural biotechnology based on several factors. These include improving the environment and human health, increasing farmer incomes, improving the nation’s food security, promoting sustainable agricultural development, and creating a more competitive position in international agricultural markets [28].

Anti-biotech efforts by non-government organizations (NGOs) in China have been active since 2010 [17, 29]. Soon after the approval of biosafety certificates of GM rice and corn, there were many misleading reports and anti-GM information presented in the public media [17], especially on the internet [29]. This misinformation may have resulted in a 5.9% rise of consumers who did not support sales of GM foods from 2007–2008 to 2010 (Fig 1A) because consumers are more likely to be influenced by an anti-biotech message than farmers. Such negative messages may have resulted in the disruption and delay of useful GM products. For example, it has been argued that GM rice and corn were not approved for commercial promotion because of increased activity of NGOs, similar to what occurred in India with Bt eggplant [30]. Although Chinese consumers may be aware of GM foods, our survey indicated that their knowledge is still limited, with only 6.3% saying that they were very familiar with GM foods (Table 1).

It should be realized that Chinese consumers’ acceptance of GM technology has not been static (Tables 2 and 4). Skepticism regarding the claims of benefits and fear of the potential risks of GM crops and foods would lead to resistance by consumers [19]. Our results suggest that educational efforts conducted by government officials, the media and esteemed Chinese scientists can facilitate the acceptance of GM technology (Fig 2, Table 1). In 2014, there were 18 million farmers in 28 countries who cultivated biotech crops on 181 million ha, with the majority of them in developing countries [14]. However, China’s decision on GM rice and maize may negatively affect decisions of other developing countries to use GM crops. Because consumers’ attitudes on GM foods is critical for decision making by government agencies, Chinese policy makers should focus on developing effective educational programs that explain the scientific facts concerning GM foods. As part of this effort, in early 2015 the state’s No. 1 Central Document pledged more government support for research on GM techniques, especially for crops. The document highlights the need for comprehensive studies to make sure that the technology is safe to use, and it also stresses that Chinese scientists must do more to convince a skeptical public of its benefits [31].

Our study demonstrated that Bt cotton farmers in China were strongly supportive of this technology (Table 3). Following this initial success with biotechnology, Bt cotton farmers will be key players in growing GM crops with increased yields and improved nutrition, which could greatly benefit food availability and improved nutrition in China. However, as these GM crops were being developed, farmers did not monitor changes in the agronomic system nor develop management strategies to handle new pest problems [32]. Our data indicated that the main driver affecting the long-term willingness to plant Bt cotton by Chinese farmers was economic benefits and not ecological considerations (Table 5), which is similar to previous survey results in European countries [18, 19]. Therefore, China should invest more on the evaluation of the safety of GM crops and foods.

China is increasing its research investments in biotechnology [16] to meet the demands and concerns of producers (for productivity-enhancing technology) and consumers (for cost savings). The attitude of Chinese scientists toward biotechnology can be an important factor for promoting the use of biotechnology and continuing the trend, which has already brought significant benefits to China [33]. Our survey of Chinese scientists showed that they have a more positive attitude concerning the development of GM crops and food in China (Fig 3) than scientists in Europe, Japan and South Korea [34–36]. The academicians who participated in this survey were from different areas of the natural sciences (Table 6). The attitude toward GM plants and foods in the Chinese scientific community should allow its members to play a significant role in promoting China's future development in this area, although different opinions still exist [29]. More research on food safety and environmental issues with GM crops and foods will help scientists make more informed judgments and conclusions.

**Table 6:** Distribution of samples and sampling methods in the survey of opinions regarding GM plants and foods in China. Data are from the study survey.

	Interviewees	Sampled regions	Number of samples	Core content
**2007–2008**				
	Farmers planting Bt cotton in China	The Yellow River Basin, The Yangtze River Basin, Xinjiang ^1^	The Yellow River Basin: 371 (50.2%); The Yangtze River Basin: 78 (10.6%); Xinjiang: 290 (39.2%)	Indicator of the farmer’s willingness to cultivate GM plants for the long-term.
	Consumers who may purchase GM foods in China	Coverage of 25 provinces or municipalities in China	Eastern Region: 840; Western Region: 169; Central Region:197; The Northeast: 210	Willingness to buy GM food.
	China's scientific community	Academicians^2^ covering all areas of natural sciences in China	254: Division of Mathematics and Physics, Chemistry, Life Sciences and Medical Sciences, Earth Sciences, Information Technology Sciences, Technological Sciences, Mechanical and Vehicle Engineering, Information and Electronic Engineering, Chemical, Metallurgical and Materials Engineering, Energy and Mining Engineering, Civil, Hydraulic and Architecture Engineering, Environment & Light and Textile Industries Engineering, Agriculture, Medical and Health, Engineering Management	Attitude on distributing GM crops and GM foods sold in the future in China.
**2010**				
	Consumers who may purchase GM foods in China	Coverage of 18 provinces or municipalities in China^3^	Eastern Region: 528; Central Region:471; Western Region: 471; The Northeast: 289	Willingness to buy GM food.

^1^ The Yellow River Basin, Yangtze River Basin and the Xinjiang region are the three largest cotton-producing areas in China. Farmers began planting Bt cotton in 1997–1998 in the Yellow River and Yangtze River Basins and approximately 5 years later in Xinjiang.

^2^ The Chinese Academy of Sciences and Chinese Academy of Engineering consisted of 719 and 699 members in 2008, and 745 and 804 in 2014, respectively. The members represent the highest level of scientific and engineering technology research in China.

^3^ These 18 provinces were included in the first sample of 25 provinces in 2007–2008. These provinces are typical representatives of the four administrative regions. The sampling locations in these 18 provinces were similar to the 2007–2008 sample, and the respondents in these areas increased in 2007–2008.

In conclusion, we surveyed the attitudes of Chinese consumers, farmers, and scientists concerning GM crops and food. Based on this study, we concluded that media coverage, as well as scientists and the government, can play a vital role in providing information regarding GM foods to the public in China. More effective and positive reports would enhance the acceptance of GM crops and food in China.

## Survey Method and Sample Description

We conducted in-person, in-house, face-to-face interviews and developed focus groups with consumers from four main geographic regions in China and farmers from the three most important cotton-producing areas of China. We also conducted a mailed survey of academicians in 15 natural science areas of China. All of the studies, including the procedure for obtaining informed consent, were approved by the China Agricultural University institutional review board.

The goals, risks and benefits of this study were explained to the interview participants, and the process for obtaining consent was also explained before the survey. All of the interview participants in this study provided consent by agreeing to schedule and participate in face-to-face interviews. Participants were asked to provide verbal consent at the beginning of the interview. Academician interviewees were invited to participate in the survey by mail. Participants in the mailed survey were informed about the goals, risks and benefits of this study, and the process for obtaining consent was also discussed in the letter. Academician interviewees provided consent by returning a survey.

### Interview surveys

We used a combination of stratified and random sampling to systematically collect samples of consumers, farmers and scientists prior to the interviews. Different demographic and socio-economic regional characteristics of China were considered in the samples as well as different cotton production regions and professional disciplines. When a pre-selected interviewee was unavailable, a replacement was chosen from the same region based on sex and age characteristics. Data used in this study were collected using a personal interview survey. A total of 4,168 people completed the questionnaires, and our research was conducted in two periods, the first period was from 2007 to 2008 and the second was in 2010. The details of the sampling are presented in Table 6.

In the first survey, a total of 1,416 participants completed responses regarding consumer's attitudes toward GM foods. They were from four main geographic regions in China (Eastern, Central, Western, Northeast), and the response rate was 87%. A total of 739 completed responses from Bt cotton growers was collected from the three most important cotton-producing areas of China (Yellow-River Basin, Yangtze-River Basin, and Xinjiang), with a response rate of 95%. The interviewees from the Chinese scientific community were members of the Chinese Academy of Science and Chinese Academy of Engineering, which covered all areas of natural sciences in China. A total of 254 completed responses of academicians were used in our analysis, with a response rate of 80%.

After biosafety certificates were approval for Bt rice and phytase corn in China, we conducted another survey from late July to early September in 2010 in similar regions to those surveyed in 2007–2008. There were 1,759 completed surveys concerning consumer’s attitudes toward GM foods, with a response rate of 98%. Because public opinion regarding GM foods is sensitive to how a question is worded, consumer’s attitudes toward GM foods were measured primarily by their willingness to buy GM foods.

### Analysis of factors influencing the choices of consumers and farmers

Two separate binary probit models [37] were used to determine other factors affecting consumers’ attitudes toward purchasing GM foods and farmer's willingness to cultivate GM plants long-term. The dependent variable used in the consumers’ survey was the respondent’s willingness to pay for plant or animal products produced through genetic modification which was defined as whether they had purchased GM foods. The response of a household was classified as 0 or 1 (1 representing purchase, 0 representing no purchase). Regression variables in the probit model regarding consumers included *Awareness of GM foods*, *Attitudes toward GM foods*, *Income*, *Gender*, *Age*, *Education level*, and *Region in China *(*Central*, *Eastern*, *Northeast*, and *Western*). The dependent variable used in the farmers' survey was the cotton farmers' long-term willing to plant Bt cotton. A value of 1 indicated that the respondent had a long-term willingness, whereas 0 indicated short-term or no willingness. Regression variables in the probit model concerning producers included *Cultivating area*, *Age*, *Proportion of Bt cotton fields of total cultivated land*, *Proportion of income from agriculture*, *Years farming*, *Gender*, *Education level*, *Family-income change because of Bt cotton*, *Pesticide change*, *Production change*, *Artificial-input change*, and *Region (Yellow-River Basin*, *Yangtze-River Basin*, *Xinjiang)*.
