# Correction: Attitudes in China about Crops and Foods Developed by Biotechnology

**DOI:** 10.1371/journal.pone.0143474

**Published:** 2015-11-18

**Authors:** Fei Han, Dingyang Zhou, Xiaoxia Liu, Jie Cheng, Qingwen Zhang, Anthony M. Shelton

The image for Fig 1 is an incorrect duplicate of Fig 3. The image that appears as Fig 3 is correct. The correct version of Fig 1 appears here.

**Fig 1:**
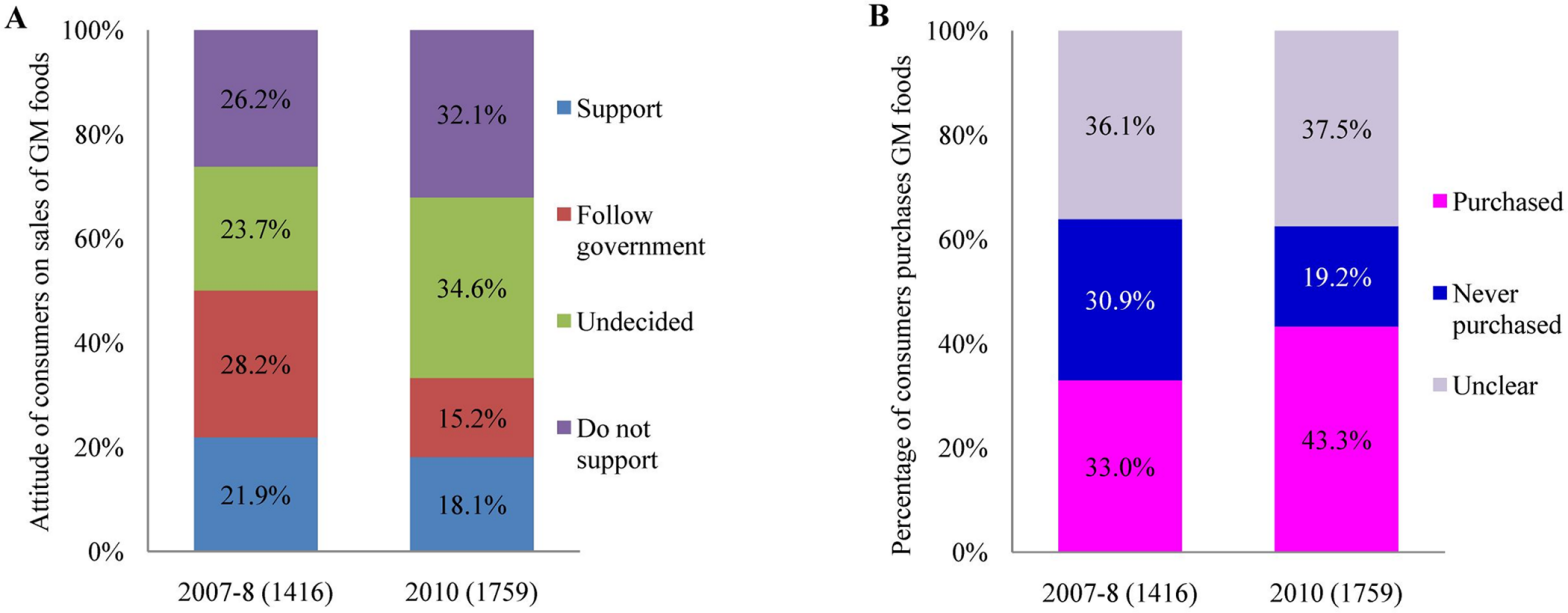
The attitudes of Chinese consumers about GM foods (2007–2008, 2010). There were 1,416 and 1,759 respondents in 2007–2008 and 2010, respectively. (A) Attitude of consumers whether GM foods should be sold, (B) Percentage of consumers who believed they had purchased GM foods.
